# Prenatal exposure to tobacco and adverse birth outcomes: effect modification by folate intake during pregnancy

**DOI:** 10.1186/s40748-022-00141-1

**Published:** 2022-09-12

**Authors:** Adrienne T. Hoyt, Anna V. Wilkinson, Peter H. Langlois, Carol E. Galeener, Nalini Ranjit, Katherine A. Sauder, Dana M. Dabelea, Brianna F. Moore

**Affiliations:** 1 Department of Health Promotion and Behavioral Sciences, UTHealth School of Public Health – Austin Regional Campus, Austin, TX USA; 2 Department of Epidemiology, UTHealth School of Public Health – Austin Regional Campus, Human Genetics, and Environmental Sciences, Austin, TX USA; 3 Department of Management, Policy and Community Health, UTHealth School of Public Health– Houston Regional Campus, Fleming Center Health for Care Management, Houston, TX USA; 4 Lifecourse Epidemiology of Adiposity and Diabetes (LEAD) CenterDepartment of PediatricsSchool of Medicine, Colorado School of Public Health, University of Colorado, Denver, USA; 5 Lifecourse Epidemiology of Adiposity and Diabetes (LEAD) Center, Colorado School of Public Health, Department of Pediatrics, Department of Epidemiology, University of Colorado School of Medicine, Colorado School of Public Health, Aurora, CO USA

**Keywords:** Smoking, Tobacco, Cotinine, Preterm Births, Small-for-gestational age births, Neonatal adiposity, Diet, Folate, Healthy start

## Abstract

**Background:**

Fetal exposure to tobacco increases the risk for many adverse birth outcomes, but whether diet mitigates these risks has yet to be explored. Here, we examined whether maternal folate intake (from foods and supplements) during pregnancy modified the association between prenatal exposure to tobacco and with preterm delivery, small-for-gestational age (SGA) births, or neonatal adiposity.

**Methods:**

Mother–child pairs (*n* = 701) from *Healthy Start* were included in this analysis. Urinary cotinine was measured at ~ 27 weeks gestation. Diet was assessed using repeated 24-h dietary recalls. Neonatal adiposity (fat mass percentage) was measured via air displacement plethysmography. Interaction was assessed by including a product term between cotinine (< / ≥ limit of detection [LOD]) and folate (< / ≥ 25^th^ percentile [1077 µg/day]) in separate logistic or linear regression models, adjusting for maternal age, race, ethnicity, education, pre-pregnancy body mass index, and infant sex.

**Results:**

Approximately 26% of women had detectable levels of cotinine. Folate intake was significantly lower among women with cotinine ≥ LOD as compared to those with cotinine < LOD (1293 µg/day vs. 1418 µg/day; *p* = 0.01). Folate modified the association between fetal exposure to tobacco with neonatal adiposity (p for interaction = 0.07) and SGA (p for interaction = 0.07). Among those with lower folate intake, fetal exposure to tobacco was associated with lower neonatal adiposity (mean difference: -2.09%; 95% CI: -3.44, -0.74) and increased SGA risk (OR: 4.99; 95% CI: 1.55, 16.14). Conversely, among those with higher folate intake, there was no difference in neonatal adiposity (mean difference: -0.17%; 95% CI: -1.13, 0.79) or SGA risk (OR: 1.15; 95% CI: 0.57, 2.31).

**Conclusions:**

Increased folate intake during pregnancy (from foods and/or supplements) may mitigate the risk of fetal growth restriction among those who are unable to quit smoking or cannot avoid secondhand smoke during pregnancy.

**Supplementary Information:**

The online version contains supplementary material available at 10.1186/s40748-022-00141-1.

## Background

Fetal exposure to tobacco (where the mother was an active or secondhand smoker) has been consistently linked to preterm delivery, [1] small-for-gestational age (SGA) at birth, [2, 3] and reduced neonatal adiposity (fat mass percentage), [4] followed by over-compensatory postnatal ‘catch up’ growth and metabolic diseases later in life. [5, 6].

Despite the steady decline in smoking rates in the United States, [7] approximately 17.3% of women of reproductive age (18–49 years) and 6.8% of pregnant women are active smokers. [8] Furthermore, ~ 35% of pregnant women are involuntarily exposed to smoke. [9] Therefore, identifying modifiable factors that may mitigate the impacts of this exposure is an important public health priority.

Of particular interest is dietary folate intake (or its synthetic form often taken as a supplement, folic acid). Beyond the well-established benefit of preventing neural tube defects, [10] evidence has been mounting that folate also may protect against various other adverse birth outcomes. [11] Furthermore, higher overall diet quality during pregnancy may lower the risk for preterm births, [12] SGA births, [13] and lower neonatal adiposity. [14] However, few published studies have examined whether maternal folate intake or overall diet quality during pregnancy may modify the associations between prenatal tobacco exposure and adverse birth outcomes.

To address this gap in knowledge, we leveraged data from *Healthy Start*, a well-characterized, racially and ethnically-diverse cohort of pregnant women and their children. We hypothesized that the associations between prenatal tobacco exposure and SGA births, preterm births, and neonatal adiposity would be stronger among those with lower folate intake and poorer overall diet quality during pregnancy.

## Methods

### Study design

Data from the *Healthy Start* cohort were utilized for this secondary data analysis. Briefly, *Healthy Start* began as a study to better understand fuel-mediated programming of offspring adiposity (NCT02273297)—although in later years expanded to explore an array of environmental and early life exposures associated with a range of infant and childhood outcomes. Study participants were pregnant women (≥ 16 years) who were patients at obstetrics clinics at the University of Colorado Hospital (< 24 weeks gestation). Exclusion criteria included: multiple gestation pregnancies; previous stillbirth or preterm birth at < 25 weeks gestation; preexisting diabetes; asthma; cancer; or psychiatric illness. Women were invited to participate in two in-person research visits during pregnancy (median: 17 and 27 weeks gestation) and one soon after delivery.

### Exposure assessment

Urinary cotinine (a metabolite of nicotine and marker of tobacco exposure [15]) was measured in a subsample of study participants at ~ 27 weeks gestation. Cotinine was analyzed in stored urine samples via solid phase competitive ELISA—with a sensitivity of 1 ng/mL (*Calbiotech Cotinine ELISA CO096D, Calbiotech, El Cajon, California*). Following a previous *Healthy Start* analysis examining similar exposures, [16] cotinine concentrations were categorized as follows: no exposure (< limit of detection [LOD]); ~ 0.05 ng/mL), maternal exposure to secondhand smoke (cotinine ≥ LOD to 550 ng/mL), and active maternal smoking (≥ 550 ng/mL; an established cut-point for active smoking [17]). Very few women were active smokers (~ 6%). Therefore, cotinine was dichotomized as no exposure (< LOD; 74%) and any exposure to tobacco (≥ LOD; 26%) to maximize power in our interaction analyses.

### Birth outcomes

Preterm births were defined as births < 37 weeks completed gestation. SGA status was estimated with sex-, race/ethnic-, and parity-specific growth curves based on the methods by Zhang and Bowes, [18] and Overpeck et al. [19] Fat mass and fat-free mass were measured within 72 h of delivery using the PEA POD (COSMED, Rome, Italy)—an air displacement plethysmography method that uses densitometric techniques to estimate fat mass from the direct measurement of mass and volume. [20] Each infant had two measurements taken with a third measurement taken if the fat mass differed by > 2.0%. The mean of the two closest measurements for each visit was utilized for analysis. Neonatal adiposity (fat mass percentage) was calculated as a proportion of the fat mass divided by total mass. [21].

### Covariates

Maternal age was calculated based on index offspring delivery date and maternal date of birth. Women self-reported maternal education and race/ethnicity via self-report on questionnaires during the 17-week (first research) visit. Maternal pre-pregnancy body mass index (BMI) was assessed as pre-pregnancy weight (kg) divided by height squared (m^2^) (pre-pregnancy weight obtained from self-report at first research visit (16.2%) or medical records (83.7%) and maternal height measured at first research visit [22]).

### The Healthy Eating Index

Maternal diet was assessed using dietary recalls conducted via the Automated Self-Administered 24-Hour Dietary Recall web-based tool during pregnancy, with a range of 1–8 dietary recalls completed by each study participant (median, 2 recalls). Diet data was processed by the Nutrition and Obesity Research Center at University of North Carolina at Chapel Hill. Maternal diet quality was ascertained via the Healthy Eating Index (HEI-2010). [22] Briefly, the HEI-2010 is a diet quality scoring system developed by the US Department of Agriculture, Center for Nutrition Policy and Promotion and the National Cancer Institute (NCI) that was designed to capture adherence of the 2010 Dietary Guidelines for Americans. The tool contains 12 components [1] total vegetables, 2) greens and beans, 3) fruit, 4) whole fruit, 5) whole grains, 6) dairy, 7) total protein foods, 8) seafood and plant proteins, 9) fatty acids, 10) sodium, 11) refined grains, and 12) empty calories) scored per 1000 kcal to give a maximum score of 100 [22] (alcohol intake was not included as all participants had < 13 g per 1000 kcal in each recall (the threshold for alcohol caloric intake inclusion [22])) and has been found to be a valid and reliable measure of diet quality. [23] The HEI-2010 values in our cohort ranged from 33–87. We assessed HEI-2010 values as both a continuous and dichotomous variable (< 61 [the median value]; ≥ 61).

### Maternal folate intake

In addition to the dietary recalls, mothers also self-reported supplement use (prenatal, multivitamin or other single nutrient) at each research visit. Folate intake from supplements was calculated by querying brand, type, and dose. [24] Participants described their supplement use within 12 weeks prior to conception (for the first pregnancy visit) or since their last visit (for mid-pregnancy and delivery visits). [25] Total maternal folate/folic acid intake was determined by combining usual daily intake of folate/folic acid from dietary sources and supplements. While the current estimated average total folate requirement for pregnant women (including both dietary and supplemental sources) is ~ 520 µg/day, [26] less than 5% of our population fell below this range. Therefore, we opted to examine folate both continuously and also categorized according to the following selected percentile cut-points (i.e. 50^th^ (< / ≥ 1384 µg/day), 25^th^ (< / ≥ 1077 µg/day), 10^th^ (< / ≥ 872 µg/day), and 5^th^ (< / ≥ 717 µg/day)).

### Statistical analysis

A linear regression model was used to examine the main effect association between prenatal tobacco exposure (no exposure, secondhand smoke exposure, and active maternal smoking) and neonatal adiposity (percent fat mass). Logistic regression models estimated the main effect association between prenatal tobacco exposure and categorical outcomes (preterm delivery and SGA births). Interaction was assessed by introducing a product term between the dichotomized cotinine variable (< LOD, ≥ LOD) and continuous or dichotomous diet variables in the separate regression models. Directed acyclic graphs (DAGS) and previous literature findings were used to determine model covariates, which included maternal age, education, race and ethnicity, pre-pregnancy BMI, and infant sex. An alpha level of 0.05 was used to determine statistical significance. As total caloric intake is also associated with each of our outcomes, we also considered maternal average daily caloric intake throughout pregnancy in each of the models as a sensitivity analysis. All statistical analyses were performed using SAS^©^ *OnDemand for Academics*.

## Results

Of the initial cohort of 1410 participants, we excluded 689 participants with missing cotinine data and 20 with missing gestational age measurements. Therefore, 701 mother–child pairs were included in the preterm birth/SGA analysis. Of these, 91 infants were missing PEA POD measurements at birth. Therefore, 630 mother–child pairs were included in the neonatal adiposity analyses. As previously described, [27] no meaningful differences in maternal or child characteristics were detected between the entire cohort and the cotinine subsample of ~ 700 mother–child pairs.

Among both analytic samples, ~ 74% of subjects had little to no cotinine exposure, ~ 20% had cotinine levels equivalent to secondhand smoke exposure, and ~ 6% had cotinine exposure in the active smoking range (Table 1). Both active smokers and those exposed to secondhand smoke tended to be younger, had a lower household income (< $40,000), lower levels of education (≤ a high school education), higher total caloric intake, lower HEI levels, and had lower mean intakes of folate/folic acid (~ 1400 µg/day among non-smokers versus ~ 1300 µg/day among smokers and those exposed to secondhand smoke).

**Table 1:** Characteristics of mother–child pairs, according to urinary cotinine in pregnancy, healthy start (2010–2014)

	Preterm & SGA Births ^**a,b**^					Neonatal Adiposity ^**f,g**^				
	Cotinine Categories ^**h**^					Cotinine Categories ^**h**^				
		< 0.05	0.05–550	≥ 550			< 0.05	0.05–550	≥ 550	
	Total (*n* = 701)	No exposure (*n* = 516, 73.6%)	Secondhand Smoke (*n* = 143, 20.4%)	Active Smoker (*n* = 42, 6.0%)	*p*-value ^**e**^	Total (*n* = 630)	No exposure (*n* = 463, 73.5%)	Secondhand Smoke (*n* = 132, 21.0%)	Active Smoker (*n* = 35, 5.5%)	*p*-value^**e**^
*Maternal characteristics *^*c*^										
Maternal age (yrs)	29 ± 6	30 ± 5	24 ± 6	27 ± 5	*p* < 0.01	28.9 ± 6	30 ± 5	24 ± 6	27 ± 5	*p* < 0.01
Pre-pregnancy BMI (kg/m2)	25 ± 6	25 ± 5	26 ± 7	26 ± 7	*p* = 0.06	25.5 ± 6	25 ± 6	26 ± 6	26 ± 7	*p* = 0.10
Gravidity, number of pregnancies	1 ± 1	1 ± 1	1 ± 1	2 ± 2	*p* < 0.01	1 ± 1	1 ± 1	1 ± 1	2 ± 2	*p* < 0.01
Maternal race/ethnicity										
non-Hispanic white	399 (57%)	337 (65%)	44 (31%)	18 (43%)	*p* < 0.01	354 (56%)	298 (64%)	42 (32%)	14 (40%)	*p* < 0.01
non-Hispanic black	84 (12%)	24 (5%)	46 (32%)	14 (33%)		75 (12%)	22 (5%)	41 (31%)	12 (34%)	
Hispanic	174 (25%)	128 (25%)	39 (27%)	7 (17%)		162 (26%)	119 (26%)	37 (28%)	6 (17%)	
Other	44 (6%)	27 (5%)	14 (10%)	3 (7%)		39 (6%)	24 (5%)	12 (9%)	3 (9%)	
Household income										
< 40,000	179 (25%)	97 (19%)	60 (42%)	22 (52%)	*p* < 0.01	165 (26%)	90 (19%)	58 (44%)	17 (49%)	*p* < 0.01
40,001–70,000	134 (19%)	105 (20%)	19 (13%)	10 (24%)		120 (19%)	94 (20%)	17 (13%)	9 (26%)	
> 70,000	264 (38%)	247 (48%)	14 (10%)	3 (7%)		234 (37%)	220 (48%)	11 (8%)	3 (9%)	
Don't know	124 (18%)	67 (13%)	50 (35%)	7 (17%)		111 (18%)	59 (13%)	46 (35%)	6 (17%)	
Mother's highest level of education										
< 12 years	87 (12%)	39 (8%)	38 (27%)	10 (24%)	*p* < 0.01	81 (13%)	36 (8%)	36 (27%)	9 (26%)	*p* < 0.01
High School Degree	111 (16%)	58 (11%)	43 (30%)	10 (24%)		101 (16%)	52 (11%)	41 (31%)	8 (23%)	
College classes or college degree	503 (72%)	419 (81%)	62 (43%)	22 (52%)		448 (71%)	375 (81%)	55 (42%)	18 (51%)	
Maternal Average Daily Total Caloric Intake Throughout Pregnancy (kcal)	2055 ± 686	2005 ± 552	2176 ± 950	2249 ± 978	*p* < 0.01	2046 ± 675	1996 ± 546	2186 ± 944	2183 ± 896	*p* < 0.01
Healthy Eating Index ^**d**^	62 ± 11	64 ± 10	56 ± 10	52 ± 9	p < 0.01	62 ± 11	64 ± 10	56 ± 10	52 ± 10	*p* < 0.01
Healthy Eating Index (≥ , < median value. 61) ^**d**^										
≥ median value	365 (52%)	316 (61%)	40 (28%)	9 (21%)	*p* < 0.01	327 (52%)	283 (61%)	36 (27%)	8 (23%)	*p* < 0.01
< median value	309 (44%)	186 (36%)	92 (64%)	31 (74%)		278 (44%)	167 (36%)	86 (65%)	25 (71%)	
missing	27 (4%)	14 (3%)	11 (8%)	2 (5%)		25 (4%)	13 (3%)	10 (8%)	2 (6%)	
Total Folic Acid Supplementation & Dietary Folate Equivalents ^**d**^	1385 ± 544	1418 ± 552	1292 ± 508	1294 ± 520	p = 0.03	1384 ± 533	1418 ± 543	1312 ± 509	1216 ± 438	*p* = 0.02
Total Folic Acid Supplementation & Dietary Folate Equivalents ^**d**^										
≥ 1077 µg/day	520 (74%)	400 (77%)	96 (67%)	24 (57%)	*p* < 0.01	470 (75%)	361 (78%)	91 (69%)	18 (51%)	*p* < 0.01
< 1077 µg/day	177 (25%)	113 (22%)	46 (32%)	18 (43%)		156 (24%)	99 (21%)	40 (30%)	17 (49%)	
missing	4 (1%)	3 (1%)	1 (1%)	0 (0%)		4 (1%)	3 (1%)	1 (1%)	0 (0%)	
*Offspring characteristics*										
Infant Sex										
Female	337 (48%)	246 (48%)	73 (51%)	18 (43%)	*p* = 0.61	309 (49%)	227 (49%)	68 (52%)	14 (40%)	*p* = 0.48
Male	362 (52%)	268 (52%)	70 (49%)	24 (57%)		321 (51%)	236 (51%)	64 (48%)	21 (60%)	
Missing	2 (0.3%)	2 (0.4%)	0 (0%)	0 (0%)		0 (0%)	0 (0%)	0 (0%)	0 (0%)	

*Note*: Continuous variables shown as mean ± standard deviations; categorical variables displayed as proportions of column totals

*SGA* small-for-gestational age {based on sex, race/ethnic and parity specific growth curves

^a^ Preterm births defined as those not completing 37 weeks gestation

^b^ 20 infants missing gestational age measurements excluded

^c^ All maternal characteristics, unless otherwise noted, were measured at 17 week pregnancy visit

^d^ Dietary characteristics collected using the automated self-administered 24-h dietary recall (ASA24) at minimum twice over the course of the pregnancy (range: 2–8 times); 25 percentile cut-point (1077mcg/day) used for categorical folate analyses

^e^ One-way analysis by variance (ANOVA) tests used to examine differences in means across the urinary cotinine categories. Chi-square square tests used to examine proportion differences across urinary cotinine categories

^f^ Neonatal adiposity (fat mass percentage) is calculated as a proportion of fat mass/total body mass (collected within 72 h following delivery using the BOD POD)

^g^ 91 infants missing neonatal adiposity measurements excluded

^h^ Contine levels expressed in nanograms/milliliter (ng/ml); limit of detection (LOD) =  ~ 0.05 ng/ml

Compared to those with high intakes of folate or high diet quality during pregnancy, mothers with lower folate intakes or lower diet quality during pregnancy were younger, had higher BMIs, were less likely to identify a non-Hispanic White, had lower household incomes, and were less educated (Supplemental Table S1). Mothers were higher folate intakes had higher caloric intakes, whereas mothers with higher diet quality during pregnancy had lower caloric intakes.

Compared to offspring with no prenatal tobacco exposure, offspring born to women with cotinine levels indicating active smoking were significantly more likely to be born SGA (aOR: 6.43; 95% CI: 2.89, 14.35) (Table 2). Prenatal tobacco exposure is associated with a slight reduction in neonatal adiposity (adjusted beta for SHS: -0.44; 95% CI: -1.30, 0.41; adjusted beta for active smoking: -0.97; 95% CI: -1.64, -0.30). Prenatal tobacco exposure was not associated with an increased risk for preterm delivery.

**Table 2:** Adjusted odds ratios and mean/beta coefficients for maternal cotinine categories and selected birth outcomes, Healthy Start (2010–2014)

aORs/aMeans for Selected Birth Outcomes ^*^							
	**Preterm Birth**		**Small-for-Gestational Age Birth**		**Neonatal Adiposity**		
	**n**_**o**_**/n**_**ō**_^†^	**aOR (95%CI)**	**n**_**o**_**/n**_**ō**_	**aOR (95%CI)**	**n**	**Adj. Beta Coefficients (95% CIs)**	**Adj. Means (95% CIs)**
*Cotinine categories*							
< 0.05 ng/mL (LOD, no exposure)	23/493	1.00 (Reference)	59/455	1.00 (Reference)	463	Reference	9.50 (8.91, 10.10)
0.05-550 ng/mL (SHS)	4/139	0.45 (0.13, 1.58)	20/123	0.92 (0.46, 1.83)	132	-0.44 (-1.30, 0.41)	9.00 (8.24, 9.76)
≥ 550 ng/mL (Active Smoking)	3/39	1.29 (0.33, 5.10)	20/22	6.43 (2.89, 14.35)	35	-0.97 (-1.64, -0.30)	7.77 (6.48, 9.07)
* p for trend*	*p* = 0.93		*p* < 0.01			*p* = 0.01	

*aORs* adjusted odds ratios, *aMean* adjusted means, *95%CIs* 95% confidence intervals

^*^All models adjusted for maternal age, education, race/ethnicity, maternal pre-pregnancy BMI, and infant sex

^†^Preterm/non-preterm births

^‡^SGA/non-SGA births

Our interaction results revealed that maternal folate intake may modify of the association between prenatal tobacco exposure with neonatal adiposity (p for interaction = 0.07) and SGA (p for interaction = 0.07) (Table 3). For instance, among those with lower folate intake (< 25^th^ percentile [< 1077 µg/day]), fetal exposure to tobacco was associated with lower neonatal adiposity (adjusted beta -2.09%; 95% CI: -3.44, -0.74) and increased SGA risk (adjusted odds ratio: 4.99; 95%CI: 1.55, 16.14). Conversely, among those with higher folate intake, there was no difference in neonatal adiposity (adjusted beta -0.17%; 95% CI: -1.13, 0.79) or SGA risk (adjusted odds ratio 1.15; 95%CI: 0.57, 2.31). A similar, albeit non-significant, pattern of interaction between prenatal tobacco exposure and folate on neonatal adiposity was noted across the 10^th^ and 5^th^ percentiles, whereas there was little evidence for interaction across the other folate cut-points (Supplemental Table S2). We found no evidence of effect modification by HEI or folate on the associations between prenatal tobacco exposure and preterm births (Table 3). Our results were similar following adjustment for prenatal daily total caloric intake (data not shown).

**Table 3:** Adjusted odds ratios and mean/beta coefficients for maternal cotinine categories and selected birth outcomes by maternal dietary factors, Healthy Start (2010–2014)

aORs/aMeans for Selected Birth Outcomes by Healthy Eating Index and Daily Folate Intake							
**Maternal Dietary Factors **^**a**^	**Preterm Birth**		**Small-for-Gestational Age Birth**		**Neonatal Adiposity**		
	**n**_**o**_**/n**_**ō**_	**aOR (95%CI)**	**n**_**o**_**/n**_**ō**_	**aOR (95%CI)**		**Adj. Beta coefficients (95% CIs)**	**Adj. Means (95% CIs)**
**Dietary Factors**							
***Healthy Eating Index (HEI)***							
*Low HEI (*< *61)*							
< 0.05 ng/mL (LOD, No Exposure)	9/177	1.00 Reference	26/159	1.00 Reference	167	Reference	9.64 (8.86, 10.43)
≥ 0.05 ng/mL (Any Smoking Exposure)	4/119	0.47 (0.12, 1.92)	29/94	1.33 (0.63, 2.81)	111	-1.03 (-2.05, -0.02)	8.69 (7.91, 9.46)
*High HEI (*≥ *61)*							
< 0.05 ng/mL (LOD, No Exposure)	14/302	1.00 Reference	32/283	1.00 Reference	283	Reference	9.32 (8.27, 10.37)
≥ 0.05 ng/mL (Any Smoking Exposure)	2/47	0.67 (0.12, 3.79)	9/40	2.11 (0.81, 5.49)	44	-0.62 (-1.98, 0.74)	8.73 (7.26, 10.20)
p for interaction term ^*^		*p* = 0.47		*p* = 0.94		*p* = 0.33	
***Total Folic Acid Supplementation & Dietary Folate Equivalents [1077 µg/day* = *25***^***th***^*** percentile]***							
< *1077 µg/day*							
< 0.05 ng/mL (LOD, No Exposure)	6/107	Reference	8/105	Reference	99	Reference	8.92 (7.85, 9.99)
≥ 0.05 ng/mL (Any Smoking Exposure)	2/62	0.32 (0.04, 2.42)	15/49	4.99 (1.55, 16.14)	57	-2.09 (-3.44, -0.74)	7.19 (6.04, 8.33)
≥ *1077 µg/day*							
< 0.05 ng/mL (LOD, No Exposure)	17/381	Reference	51/347	Reference	361	Reference	9.71 (8.98, 10.43)
≥ 0.05 ng/mL (Any Smoking Exposure)	5/115	0.94 (0.28, 3.14)	25/95	1.15 (0.57, 2.31)	109	-0.17 (-1.13, 0.79)	9.43 (8.57, 10.29)
p for interaction term *		*p* = 0.63		*p* = 0.07		*p* = 0.07	

aORs = adjusted odds ratios; aMean = adjusted means; 95%CIs = 95% confidence intervals

SGA = small-for-gestational age {based on sex, race/ethnic and parity specific growth curves (add citations here)

^*^ *P*-values for interaction generated by adding product terms between maternal cotinine and nutrient categories (continuous form) in separate models

^a^ All models adjusted for maternal age, education, race/ethnicity, maternal pre-pregnancy BMI, and infant sex

## Discussion

Our findings suggest that dietary folate minimizes the risk of tobacco-induced fetal growth restriction. By contrast, overall diet quality during pregnancy did not modify the risk for adverse birth outcomes. Given that many pregnant women are unable to successfully quit smoking [28] or are involuntarily exposed to SHS [29], our findings may have important public health implications for mitigating risks associated with this exposure.

Our interaction findings may provide insights about the mechanisms underlying the associations between prenatal tobacco exposure and systematic growth restriction. One key pathway may involve maternal and fetal oxidative stress. Tobacco is known to increase markers of oxidative stress in the placenta via specific epigenomic modulations in key metabolic pathways. [30] Conversely, folate exhibits antioxidant [31] and anti-inflammatory properties [32]which may independently improve fetal growth [31].

A more novel mechanistic pathway may involve tobacco-induced disruption of the homocysteine-methionine cycle of the fetus, [33] which is associated with deficiencies in circulating folate. Tobacco use during pregnancy is known to increase maternal homocysteine [34], which is associated with impaired uteroplacental blood flow and fetal growth restriction. [35] These effects may be offset by maintaining adequate dietary folate intake. [36] This hypothesis is supported by the work of Bakker and colleagues, [37] who reported that the combination of prenatal tobacco exposure and higher maternal homocysteine concentrations is associated with lower birth weight, but not preterm delivery.

Our interaction findings may have the potential for a large and immediate public health impact. Pregnant women have a heightened interest in dietary information [38] and are particularly receptive to dietary counseling during prenatal care. [39] Pregnant women are already encouraged to consume adequate folate to reduce the risk for neural tube defects, [10] and most do. [24] Overall, our findings support the positive impacts of maternal folate intakes during pregnancy on improving fetal growth.

Contrary to previous studies [40, 41], we found no evidence that overall diet quality mitigated the risk of prenatal tobacco exposure on the adverse birth outcomes. [40, 41]There are several factors that could explain this discrepancy. First, diet quality was captured via a Mediterranean diet score in previous studies [40, 41], whereas we utilized the Healthy Eating Index. Second, our study population had a relatively high diet quality as compared to the national population (median HEI in our study population was 61, whereas data from the National Health and Nutrition Examination Survey reports a median HEI of 52 [42]. Finally, our interaction findings with folate intake provides points to specific mechanisms (e.g. homocysteine pathways) that may not be reflected in measures of overall diet quality.

One limitation of our approach is the one-time measurement of cotinine. This prohibited our ability to examine trimester-specific effects, which have been noted in previous studies. [43] Additionally, since cotinine has a relatively short half-life [44], our one-time assessment may not be an accurate representation of tobacco exposure throughout pregnancy. [44] Our categorization of cotinine (< / > 550 mg/mL) is a highly sensitive but less specific cut-point for distinguishing active smokers from passive smokers. This may have resulted in some exposure misclassification. We speculate that the exposure misclassification would nondifferential with respect to the outcome, thus our effect estimates would be biased towards the null.

While many maternal-infant factors were controlled for in our analyses, residual confounding cannot be ruled out. Additionally, the small number of offspring born preterm or SGA births may have hindered our ability to detect an interaction between tobacco and diet. Lastly, our ability to generalize findings to other populations is limited, as mothers in our cohort may have had higher education levels, higher diet quality during pregnancy, and lower BMI levels than the general population.

Some of the novel aspects of the study include our ability to limit information bias. First, we utilized air displacement plethysmography, which has been shown to be a convenient, reliable, and valid method for measuring neonatal body composition. [20] With respect to nutrition, maternal folate intake was determined by both dietary recalls and self-reported supplement use, which provides a more complete picture of folate consumed during pregnancy than relying on dietary recall alone. Additionally, our use of repeated dietary recalls may have minimized recall bias [25] by giving participants more than one opportunity to report previously forgotten food items and estimate portion sizes. [45] Yet, there is still some potential for measurement error, since most of the women in our study completed only two dietary recalls, and may have misrepresented or completely omitted the amounts of certain foods/beverages consumed. [46, 25, 22, 47].

## Conclusions

Despite the widely communicated risks of smoking during pregnancy, many pregnant women smoke or are involuntarily exposed to SHS. [48] This is concerning, given the well-documented associations between prenatal tobacco exposure on systematic growth restriction of the fetus. There is a need to identify modifiable factors, such as folate intake during pregnancy (increased via diet or folic acid supplementation), that may protect the fetus against these environmental insults. Our results suggest that higher levels of folate intake during pregnancy (≥ 1077 µg/day) may limit the effects of prenatal tobacco exposure on systematic growth restriction. Our findings point to increasing overall folate intake as a potential mitigation strategy among pregnant women who are unable to avoid SHS or quit smoking during pregnancy.

## Supplementary Information

**Additional file 1:** **Supplemental Table S1. **Maternal characteristics, according to supplementation intake and dietary factors, Healthy Start (2010-2014). **Supplemental Table S2.** Adjusted odds ratios and mean/beta coefficients for maternal cotinine categories and selected birth outcomes by maternal total dietary folate intake, Healthy Start (2010-2014).

## Data Availability

The dataset analyzed in this study is protected under an institution review board protocol and is not available for distribution.

## Abbreviations

ABO: Adverse birth outcome
ANOVA: One-way analysis by variance
BMI: Body Mass Index
DAG: Directed acyclic graphs
FFM: Fat-free mass
FM: Fat mass
HEI: Healthy Eating Index
LOD: Limit of detection
NCI: National Cancer Institute
SGA: Small-for-gestational age
SHS: Secondhand smoke
