# Supplementary material for: Prenatal exposure to tobacco and adverse birth outcomes: effect modification by folate intake during pregnancy
Source: Matern Health Neonatol Perinatol. 2022 Sep 12;8:6. doi: 10.1186/s40748-022-00141-1 (PMC9465971; doi:10.1186/s40748-022-00141-1)
Supplement: Supplementary file 1 — Additional file 1: Supplemental Table S1. Maternal characteristics, according to supplementation intake and dietary factors, Healthy Start (2010-2014). Supplemental Table S2. Adjusted odds ratios and mean/beta coefficients for maternal cotinine categories and selected birth outcomes by maternal total dietary folate intake, Healthy Start (2010-2014). [file 40748_2022_141_MOESM1_ESM.docx]

|  |  |  |  |  |  |  |  |  |  |  |
| --- | --- | --- | --- | --- | --- | --- | --- | --- | --- | --- |
| **Supplemental Table S1.** Maternal characteristics, according to supplementation intake and dietary factors, Healthy Start (2010-2014) | | | | | | | | |  |  |
|  |  |  |  |  |  |  |  |  |  |  |
|  |  |  |  |  |  |  |  |  |  |  |
|  | | | **Total Folic Acid Supplementation & Dietary Folate Equivalents**^a^ | | | **Healthy Eating Index (<, ≥ median value. 61)**^a^ | | |  |  |
|  | | | **<1077µg/day** | **≥1077µg/day** | **p-value**^c^ | **< median value** | **≥ median value** | **p-value**^c^ |  |  |
| *Maternal characteristics^b^* | | |  |  |  |  |  |  |  |  |
| **Maternal Age (yrs)** | | | 28 ± 6 | 29 ± 6 | p=0.02 | 27 ± 6 | 31 ± 5 | p < 0.01 |  |  |
| **Pre-Pregnancy BMI (kg/m^2^)** | | | 26 ± 6 | 25 ± 6 | p=0.08 | 26 ± 6 | 24 ± 5 | p < 0.01 |  |  |
| **Maternal race/ethnicity** | | |  |  |  |  |  |  |  |  |
| non-Hispanic white | | | 78 (45%) | 317 (61%) | p<0.01 | 129 (42%) | 257 (70%) | p<0.01 |  |  |
| non-Hispanic black | | | 22 (13%) | 62 (12%) |  | 58 (19%) | 20 (5%) |  |  |  |
| Hispanic | | | 62 (35%) | 112 (21%) |  | 95 (31%) | 71 (19%) |  |  |  |
| Other | | | 13 (7%) | 31 (6%) |  | 27 (9%) | 17 (5%) |  |  |  |
| **Household income** | | |  |  |  |  |  |  |  |  |
| <40,000 | | | 52 (30%) | 126 (24%) | p<0.01 | 106 (34%) | 67 (18%) | p<0.01 |  |  |
| 40,001-70,000 | | | 40 (23%) | 93 (18%) |  | 56 (18%) | 72 (20%) |  |  |  |
| >70,000 | | | 42 (24%) | 220 (42%) |  | 69 (22%) | 189 (52%) |  |  |  |
| Missing/Don't know | | | 41 (23%) | 83 (16%) |  | 78 (25%) | 37 (10%) |  |  |  |
| **Mother's highest level of education** | | |  |  |  |  |  |  |  |  |
| <12 years | | | 33 (19%) | 54 (10%) | p<0.01 | 64 (21%) | 18 (5%) | p<0.01 |  |  |
| High School Degree | | | 33 (19%) | 77 (15%) |  | 70 (23%) | 35 (10%) |  |  |  |
| College classes or college degree | | | 109 (62%) | 391 (75%) |  | 175 (57%) | 312 (85%) |  |  |  |
| **Maternal Average Daily Total Caloric Intake Throughout Pregnancy (kcal)** | | | 1745 ± 613 | 2158 ± 678 | p<0.01 | 2090 ± 732 | 1989 ± 538 | p=0.04 |  |  |
|  | | |  |  |  |  |  |  |  |  |
| ^a^ Dietary characteristics collected using the automated self-administered 24-hour dietary recall (ASA24) at minimum twice over the course of the pregnancy (range: 2-8 times); 25 percentile cut-point (1077mcg/day) used for categorical folate analyses. | | | | | | | | |  |  |
| ^b^ All maternal characteristics, unless otherwise noted, were measured at 17 week pregnancy visit. | | | | | | | | |  |  |
| ^c^ Independent samples t-tests used to assess differences in means across cotinine categories for continuous variables (means +/- standard deviations). Chi-square square tests used to examine proportion differences across urinary cotinine categories. | | | | | | | | |  |  |

| **Supplemental Table S2.** Adjusted odds ratios and mean/beta coefficients for maternal cotinine categories and selected birth outcomes by maternal total dietary folate intake, Healthy Start (2010-2014) | | | | | | | |  |  |  |
| --- | --- | --- | --- | --- | --- | --- | --- | --- | --- | --- |
|  |  |  |  |  |  |  |  |  |  |  |
| **aORs/aMeans for Selected Birth Outcomes by Healthy Eating Index and Daily Folate Intake** | | | | | | | |  |  |  |
| **Total Maternal Folate^a^** | **Preterm Birth** | | **Small-for-Gestational Age Birth** | | **Neonatal Adiposity** | | |  |  |  |
|  | **n_o_/n_ō_** | **aOR (95%CI)** | **n_o_/n_ō_** | **aOR (95%CI)** |  | **Adj. Beta coefficients (95% CIs)** | **Adj. Means (95% CIs)** |  |  |  |
|  |  |  |  |  |  |  |  |  |  |  |
| ***Total Folic Acid Supplementation & Dietary Folate Equivalents*** |  |  |  |  |  |  |  |  |  |  |
| <0.05ng/mL (LOD, No Exposure) | 23/488 | 1.00 Reference | 59/452 | 1.00 Reference | 463 | 1.00 Reference | 9.48 (8.88, 10.08) |  |  |  |
| ≥0.05ng/mL (Any Smoking Exposure) | 7/178 | 0.61 (0.22, 1.68) | 40/144 | 1.75 (0.99, 3.08) | 167 | -0.76 (-1.54-0.03) | 8.73 (8.03, 9.42) |  |  |  |
| *p for interaction term** |  | p=0.74 |  | p=0.98 |  | p=0.29 | |  |  |  |
|  |  |  |  |  |  |  |  |  |  |  |
| ***Total Folic Acid Supplementation & Dietary Folate Equivalents [1304µg/day=50^th^ percentile]*** |  |  |  |  |  |  |  |  |  |  |
| *<1304µg/day* |  |  |  |  |  |  |  |  |  |  |
| <0.05ng/mL (LOD, No Exposure) | 11/232 | Reference | 25/218 | Reference | 216 | Reference | 9.00 (8.16, 9.83) |  |  |  |
| ≥0.05ng/mL (Any Smoking Exposure) | 4/104 | 0.44 (0.12, 1.69) | 24/84 | 2.32 (1.09, 4.95) | 97 | -0.81 (-1.88, 0.26) | 8.20 (7.27, 9.13) |  |  |  |
|  |  |  |  |  |  |  |  |  |  |  |
| ≥*1304µg/day* |  |  |  |  |  |  |  |  |  |  |
| <0.05ng/mL (LOD, No Exposure) | 12/256 | Reference | 34/234 | Reference | 244 | Reference | 10.17 (9.27, 11.08) |  |  |  |
| ≥0.05ng/mL (Any Smoking Exposure) | 3/73 | 1.17 (0.23, 5.98) | 16/60 | 1.29 (0.53, 3.18) | 69 | -0.84 (-1.99, 0.31) | 9.27 (8.49, 10.08) |  |  |  |
| *p for interaction term** |  | p-0.97 |  | p=0.98 |  | p=0.49 | |  |  |  |
| ***Total Folic Acid Supplementation & Dietary Folate Equivalents [872µg/day=10^th^percentile]*** |  |  |  |  |  |  |  |  |  |  |
| *<872µg/day* |  |  |  |  |  |  |  |  |  |  |
| <0.05ng/mL (LOD, No Exposure) | 5/36 | Reference | 4/37 | Reference | 35 | Reference | 9.74 (8.08, 11.39) |  |  |  |
| ≥0.05ng/mL (Any Smoking Exposure) | 1/31 | N/A | 6/26 | 1.32 (0.13, 13.62) | 28 | -2.82 (-4.68, -0.96) | 7.40 (5.93, 8.88) |  |  |  |
|  |  |  |  |  |  |  |  |  |  |  |
| ≥*872µg/day* |  |  |  |  |  |  |  |  |  |  |
| <0.05ng/mL (LOD, No Exposure) | 18/452 | Reference | 55/415 | Reference | 425 | Reference | 9.56 (8.90, 10.21) |  |  |  |
| ≥0.05ng/mL (Any Smoking Exposure) | 6/146 | 1.00 (0.33, 3.05) | 34/118 | 1.70 (0.93,3.13) | 138 | -0.31 (-1.18, 0.55) | 9.19 (8.43, 9.96) |  |  |  |
| *p for interaction term** |  | p=0.23 |  | p=0.83 |  | p=0.12 | |  |  |  |
|  |  |  |  |  |  |  |  |  |  |  |
| ***Total Folic Acid Supplementation & Dietary Folate Equivalents [717µg/day=5^th^percentile]*** |  |  |  |  |  |  |  |  |  |  |
| <717µg/day |  |  |  |  |  |  |  |  |  |  |
| <0.05ng/mL (LOD, No Exposure) | 3/18 | Reference | 1/20 | Reference | 17 | Reference | 9.15 (6.85, 11.44) |  |  |  |
| ≥0.05ng/mL (Any Smoking Exposure) | 0/17 | N/A | 3/14 | N/A | 14 | -3.59 (-6.64, -0.55) | 6.35 (4.57, 8.13) |  |  |  |
|  |  |  |  |  |  |  |  |  |  |  |
| ≥717µg/day |  |  |  |  |  |  |  |  |  |  |
| <0.05ng/mL (LOD, No Exposure) | 20/470 | Reference | 58/432 | Reference | 443 | Reference | 9.54 (8.91, 10.16) |  |  |  |
| ≥0.05ng/mL (Any Smoking Exposure) | 7/160 | 0.89 (0.31, 2.54) | 37/130 | 1.69 (0.94, 3.03) | 152 | -0.47 (-1.28, 0.35) | 9.03 (8.30, 9.76) |  |  |  |
| *p for interaction term** |  | p=0.95 |  | p=0.58 |  | p=0.15 | |  |  |  |
|  |  |  |  |  |  |  |  |  |  |  |
|  |  |  |  |  |  |  |  |  |  |  |
| aORs=adjusted odds ratios; aMean=adjusted means; 95%CIs=95% confidence intervals | | | | | | | |  |  |  |
| SGA=small-for-gestational age {based on sex, race/ethnic and parity specific growth curves (add citations here) | | | | | | | |  |  |  |
| * P-values for interaction generated by adding product terms between maternal cotinine and nutrient categories (continuous form) in separate models. | | | | | | | |  |  |  |
| ^a^ All models adjusted for maternal age, education, race/ethnicity, infant sex and maternal pre-pregnancy BMI. | | | | | | | |  |  |  |
